# On the Difficulty to Detect Carbapenem Resistance in the Environment: Characterisation of *Escherichia coli* With Reduced Carbapenem Susceptibility Isolated in a French River

**DOI:** 10.1111/1758-2229.70162

**Published:** 2025-07-21

**Authors:** Laëtitia Le Devendec, Mai‐Lan Tran, Eric Jouy, Fabien Vorimore, Amandine Wilhelm, Benoit Gassilloud, Patrick Fach, Sandrine Baron, Sabine Delannoy

**Affiliations:** ^1^ Mycoplasmology‐Bacteriology and Antimicrobial Resistance Unit, French Agency for Food, Environmental and Occupational Health & Safety (Anses), Ploufragan‐Plouzané‐Niort Laboratory Ploufragan France; ^2^ Pathogenic E. coli Unit, French Agency for Food, Environmental and Occupational Health & Safety (Anses), Laboratory for Food Safety Maisons‐Alfort France; ^3^ IdentyPath Genomics Platform, French Agency for Food, Environmental and Occupational Health & Safety (Anses), Laboratory for Food Safety Maisons‐Alfort France; ^4^ Laboratory for Hydrology, French Agency for Food, Environmental and Occupational Health and Safety (Anses) Nancy France

**Keywords:** antimicrobial resistance, carbapenems, *E. coli*, environment, freshwater

## Abstract

During an 18‐month longitudinal study, bi‐monthly water samples were taken upstream and downstream of a watershed. In order to detect carbapenem‐resistant *E. coli* , the CHROMIDCarba medium was used. Of the 863 isolates collected from 144 samples, *E. coli* identification was confirmed for only seven of them, isolated on the same day. For six isolates, a slightly reduced susceptibility to carbapenems was observed. The results of the whole genome sequencing indicate that the six isolates belong to the same clone (O8:H7, ST196). Furthermore, a mutation of the *ompC* porin coupled with the presence of the *bla*_CMY‐2_ gene, on an *IncI1* plasmid, would be at the origin of the reduced sensitivity of these strains to carbapenems. This type of mechanism has already been described in human clinical cases. To our knowledge, this is the first time that it has been identified in strains from the aquatic environment. The detection of *E. coli* with reduced susceptibility to carbapenems one time in 18 months (one out of 36 sampling dates) could be considered a one‐time event. However, this illustrates the importance of monitoring the aquatic environment but also the methodological difficulties of such surveillance due to the poor efficacy of the isolation method.

## Graphical abstract

We characterised *E. coli* strains with reduced carbapenem susceptibility isolated from a rural watershed. Of the 863 isolates collected on ChromID Carba medium, *E. coli* identification was confirmed for only 7 isolates, 6 of which show reduced susceptibility to carbapenems. Whole genome sequencing indicated that a mutation of the *ompC* porin coupled with the presence of the *bla*_CMY‐2_ gene, on an *IncI1* plasmid, would be at the origin of the reduced sensitivity of these strains to carbapenems.

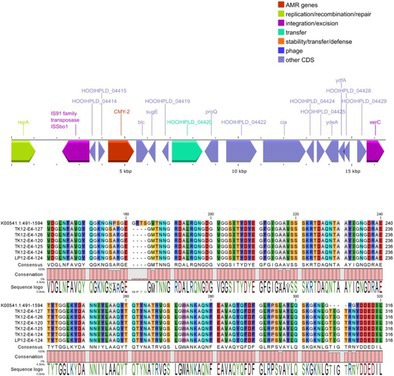

## 1 Introduction

Antimicrobial resistance (AMR) is one of the main challenges of the ‘One health’ initiative, and the environment is increasingly recognised as an important contributing compartment to the AMR problem, together with humans and animals. AMR is a natural phenomenon exacerbated by anthropogenic activity. Initially mainly observed in hospital environments, AMR has spread to all ecosystems impacted by human activities mainly through wastewater discharge, including rivers downstream of cities, groundwater, farm animals and wildlife. As all these environments and ecosystems are interconnected, it is important to better understand the spread of resistance.

AMR is genetically encoded and, if not intrinsic, can be due either to mutations in endogenous genes (Coculescu 2009) or to the horizontal acquisition of foreign resistance genes (Blair et al. 2015) carried by mobile genetic elements such as plasmids, integrons, transposons or bacteriophages. AMR is therefore not limited to a particular species or host. It can spread from commensal to pathogenic bacteria, from animal to human and vice versa, directly through the propagation of resistance genes between bacteria via these mobile genetic elements (Peterson and Kaur 2018). The spread of resistance genes via mobile genetic elements can take on an epidemic scale. A well‐documented example of such a phenomenon is the mobilisation of the chromosomal β‐lactamase *ampC* gene on a plasmid, leading to its increased expression and global spread (Haenni, Châtre, and Madec 2014). All bacteria can thus be considered a potential reservoir of resistance genes that can be transferred to other pathogenic or commensal bacteria in animals, humans and the environment (Argudín et al. 2017; Perreten et al. 2005; Manaia 2017; Torres et al. 2018; McMillan et al. 2019). Bacteria and genes cross environments and species boundaries, and it is thus necessary to take into account all compartments (humans, animals and the environment) to understand the dynamics of AMR and identify emerging trends outside of the clinical setting. In addition to determining the prevalence of resistant bacteria, it is therefore important to identify the mechanisms involved in resistance and to monitor their dissemination in humans, animals and ecosystems. However, surveillance systems outside the healthcare settings are scarce, and they are usually mainly centred on specific food‐producing animals. The United Nations Environment Program highlights the fact that the role of the environment in the emergence and spread of antibiotics resistance has not received enough attention so far (UNEP 2023).

Surface waters capture biological material from the entire community (Tiwari et al. 2022) and are emerging as potential AMR hotspots (Kunhikannan et al. 2021; Reddy et al. 2022). They are the receiving bodies of various AMR sources such as wastewater treatment plant effluents, combined sewer overflows, runoff from agricultural surfaces, aquaculture, landfill leachates or faecal matter of human and animal origin (Liguori et al. 2022), combined with the presence of multiple antibiotic residues and other chemical substances (pesticide, heavy metals) from various anthropogenic sources, including hospital effluents, industrial and agricultural runoff (Huijbers et al. 2015). Incoming bacteria, which are not autochthonous to the aquatic environment, are largely eliminated because they are not adapted to environmental conditions. This transient environmental persistence may nevertheless allow the transfer of their resistance genes to the rich flora of environmental bacteria through mechanisms involving mobile genetic elements. Because they are better adapted to their environment than the contaminating bacteria (or allochthonous bacteria), autochthonous bacteria that have become resistant (or less susceptible) can then form an environmental reservoir of resistance genes, with a risk of spreading to humans or animals in the event of new exposure, for example through drinking water (Shao et al. 2018), crop irrigation (Murray et al. 2019) or recreational aquatic activities. It is therefore important to better characterise the role of aquatic ecosystems in the dispersal of antibiotic resistance genes in the environment.

Carbapenems are broad‐spectrum beta‐lactam antimicrobials considered as last resort for the treatment of severe, life‐threatening infections and critically important antimicrobials for human medicine (Harding‐Crooks et al. 2023; WHO 2019). However, the widespread use of these antibiotics contributed to the emergence of carbapenem‐resistant bacteria (Albiger et al. 2015; Iovleva and Doi 2017), and many studies have revealed that resistance to carbapenems is increasing throughout the world (Morrison and Rubin 2015; Grundmann et al. 2017; Magiorakos et al. 2017; ECDC and WHO 2023). Carbapenem resistance in Enterobacterales is primarily mediated through the production of carbapenemases, β‐lactamase enzymes capable of hydrolysing carbapenems, such as KPC (Class A), NDM, VIM and IMP (Class B metallo‐β‐lactamases), and OXA‐48‐like enzymes (Class D). In addition to enzymatic degradation, resistance may arise from non‐enzymatic mechanisms, including overexpression of efflux pumps, loss or alteration of outer membrane porins and hyperproduction of extended‐spectrum β‐lactamases (ESBLs) or AmpC enzymes. While these mechanisms alone typically confer low‐level resistance, they can significantly elevate resistance when acting synergistically, particularly in the presence of β‐lactamases (Simner et al. 2024). The frequent localisation of carbapenemase genes on mobile genetic elements further accelerates dissemination across bacterial populations. Carbapenem‐resistant Enterobacterales (CRE) have thus been identified as critical priority pathogens (WHO 2024) and their spread, in particular that of plasmid‐mediated resistance mechanisms, needs to be closely monitored.

Current methods for assessing the microbiological quality of surface water and wastewater are primarily based on the most probable number (MPN) approach, which estimates the concentration of faecal indicator bacteria without yielding isolates for further characterisation (European Parliament 2014; ISO 2014a). As no standardised protocols currently exist for the isolation of *Escherichia coli* from surface waters, we sought to determine whether the sampling framework used for drinking water microbiological monitoring (WHO 2022; European Parliament 2020)–particularly for indicators of faecal contamination– specifically membrane filtration of up to 100 mL volumes, as outlined in ISO 9308‐1 (ISO 2014b), could be adapted for the detection of CRE in surface water. Although these standards were developed for water intended for human consumption, where low bacterial contamination is expected, they provide a practical framework for obtaining isolates under field conditions, particularly in the absence of alternative guidance.

While *K. pneumoniae* is a well‐established clinical reservoir of carbapenem resistance, *E. coli* serves as a key indicator organism for understanding the broader dissemination of resistance genes across clinical, environmental and agricultural domains due to its ubiquity, genetic diversity and ecological versatility. We therefore chose to focus on *E. coli* as a sentinel organism for tracking carbapenem resistance in the environment. Acknowledging the inherent limitations of small‐volume sampling for detecting low‐abundance targets, our objective was not to capture the full diversity or prevalence of CRE, but rather to assess the feasibility of identifying contamination events using standard microbiological water surveillance procedures. This study therefore aims to explore the potential of integrating AMR monitoring—specifically for CRE—into existing water quality surveillance systems. Subsequently, we characterised *E. coli* strains with reduced carbapenem susceptibility isolated from a rural watershed receiving effluents from three wastewater treatment plants and influenced by surrounding agricultural and fish farming activities.

## 2 Material and Methods

### 2.1 Sampling

A river located in the western part of France (Britany) was sampled twice a month for 18 months from February 2017 to July 2018. Water grab samples (5 L) were collected at four stations from the source to the mouth of the river. The upstream sampling site is located approximately 3.5 km from the source of the river, with 7.5 km separating the most extreme sampling points. There is no hospital upstream of the sampling sites. Two wastewater treatment plants treating domestic wastewater are located on tributaries feeding the studied watershed between the sampling points. However, there is significant agricultural pressure throughout the catchment area. Water samples were stored at 4°C until processed. Processing was performed within 4 h.

### 2.2 Bacterial Isolates

For each of the 144 samples collected, three volumes (100, 10 and 1 mL) were filtered onto 0.45 μm cellulose ester membranes (Millipore, Watford, UK). The membranes were transferred onto ChromID CARBA agar plates (Biomerieux, Marcy l'Etoile, France). Agar plates were incubated for 24 h at 37°C and examined for pink to burgundy colonies indicative of Carbapenemase producing *E. coli* . Up to 10 characteristic colonies were picked per plate and purified on ChromAgar Orientation plates (Biomerieux, Marcy l'Etoile, France). Purified isolates were stored at −20°C in Brain Heart Broth with 20% glycerol.

Putative Carbapenemase‐producing *E. coli* were identified using a Microflex LT mass spectrometer and a Sirius spectrometer Biotyper (Bruker Daltonics, Germany) with FlexControl V3.0 software and the MBT Compass reference library, versions 2019 (MaldiBiotyperDBUpdate_V7.0.0.0) to 2022 (MaldiBiotyperDBUpdate_V10.0.0.0). Bacterial cells were grown for 24 h on Mueller Hinton Agar at 37°C before analysis. Two different sample preparation procedures were used on three colonies per strain: (i) direct transfer (spotting) of the colony onto a target plate, (ii) the formic acid overlay method that consists of depositing 1 μL of formic acid on direct colony spotting, according to the manufacturer's recommendations. After drying, each spot was overlaid with 1 μL of 10 mg/mL of α‐cyano‐4‐hydroxycinnamic acid (HCCA) matrix solution (Bruker Daltonics). The identification criteria used were those recommended by the manufacturer. Log scores ≥ 2 were considered reliable for species identifications, log scores ≥ 1.7 and < 2.0 were defined as reliable for genus identification and log scores < 1.7 as non‐reliable identification.

### 2.3 Antimicrobial Susceptibility Testing

The minimal inhibitory concentration for the confirmed *E. coli* isolates was determined by the microdilution broth method using standardised EUVSEC and EUVSEC2 microplates (Thermofisher) according to the EFSA protocol. The 20 antimicrobial agents tested belong to nine classes: beta‐lactams including three sub‐classes: penicillins (ampicillin [AMP], and temocillin [TRM]), cephalosporins (cefotaxime [FOT], ceftazidime [TAZ], cefoxitin [FOX] and cefepime [FEP]) and carbapenems (meropenem [MERO], ertapenem [ETP] and imipenem [IMI]), quinolones (ciprofloxacin [CIP] and nalidixic acid [NAL]), tetracyclines (tetracycline [TET]), inhibitors of the folate pathway (trimethoprim [TMP] and sulfamethoxazole [SMX]), aminoglycosides (gentamicin [GEN]), macrolides (azithromycin [AZI]), glycylcycline (tigecycline [TGC]), polymyxins (colistin [COL]) and amphenicols (chloramphenicol [CHL]). *E. coli* ATCC 25922 was used as a control strain.

Interpretation criteria used for the MIC value are those defined by Eucast (Eucast 2024). Both epidemiological cut‐off value (Ecoff) and clinical breakpoint were used. Ecoff is used to separate bacterial populations on the basis of MIC distribution data into wild type isolate (WT) and non wild type isolate (NWT). WT isolate is defined by the absence of acquired and mutational resistance mechanisms to the drug in question. A NWT microorganism is defined for a species by the presence of an acquired or mutational resistance to the drug in question. The clinical breakpoint is reserved for the prediction of clinical efficacy (Silley 2012).

### 2.4 DNA Extraction for Sequencing

Prior to genomic DNA isolation for Illumina and MinION sequencing, each strain was cultivated overnight at 37°C in brain heart infusion (BHI). Genomic DNA was prepared from 1 mL of BHI overnight cultures using the Blood and tissue kit (Qiagen) according to the manufacturer's instructions.

### 2.5 Illumina MiSeq Sequencing

Libraries were prepared from 1 ng of gDNA using the Nextera XT DNA Library Preparation Kit (Illumina Inc.) following the manufacturer's instructions. Libraries were sequenced using the MiSeq Reagent Kit v2 (2 × 150 bp) (Illumina Inc.) on a MiSeq System.

### 2.6 ONT MinION Sequencing

The MinION library was prepared with 150 ng DNA using the rapid barcoding kit (SQK‐RBK004, Oxford Nanopore Technologies) according to the manufacturer's instructions. Long‐read sequencing was performed using a Flongle (R9.4.1) flow cell on the Oxford Nanopore MinION sequencer for 24 h.

### 2.7 Sequencing Data Analysis

The raw Illumina reads were trimmed (minimum length, 35 bp; quality score, 0.05) and assembled in CLC Genomics Workbench v21.0.5 (Qiagen) by de novo assembly (minimum contig length, 1000 bp).

The serotype of the isolates as well as the presence of resistance genes and plasmid replicons were determined using Genial v1.0 (an abricate v0.8.7 wrapper; https://github.com/p‐barbet/GENIAL) and the Serotypefinder, Resfinder and Plasmidfinder databases, respectively. Parameters of 80% sequence identity with a minimum sequence overlap of 60% were used [‐‐min‐cov 60 ‐‐min‐id 80]. MLST and virulence factors were determined using MLST v2.0 (with the *E. coli* #1 MLST scheme) and Virulence Finder v2.0.3 respectively (database version 2022‐12‐02) on the CGE website (Wirth et al. 2006; Larsen et al. 2012; Joensen et al. 2014; Malberg Tetzschner et al. 2020). The phylogroup was determined using the online Clermontyping tool (http://clermontyping.iame‐research.center/).

To assess the genetic relatedness between the isolates, we downloaded all ST196 genome assemblies with corresponding metadata available from Enterobase (accession date: 11 July 2023; *n* = 97). Core genome MLST (cgMLST) analysis was performed using chewBBACA v.2.8.5 and the Innuendo *E. coli* cgMLST schema (2360 loci) (Silva et al. 2018; Alikhan et al. 2018). *E. coli* Sakai (accession # BA000007) was used as an outgroup. A custom python script (File S1) was used to remove samples with < 90% of alleles identified and remove loci identified in < 90% of samples. The ExtractCgMLST module of chewBBACA was used to replace all non‐numerical values in the resulting table with 0. A neighbour‐joining tree was generated from the cgMLST profiles using grapetree (https://github.com/achtman‐lab/GrapeTree; Zhou et al. 2018) and displayed using FigTree v1.4.4 (https://github.com/rambaut/figtree). Differences between cgMLST profiles were calculated using cgmlst‐dists (https://github.com/tseemann/cgmlst‐dists). The Average Nucleotide Identity (ANI) was calculated using FastANI v1.33 (https://github.com/ParBLiSS/FastANI).

Basecalling of the raw MinION fast5 data was performed with Guppy basecaller version 4.4.2. Hybrid assembly using MiSeq and MinION reads was performed using Unicycler version 0.4.8 [‐‐min_fasta_length 1000]. The assembled genome was annotated with Prokka v1.14.6.

The pMLST profile was determined using pMLST 2.0 with IncI1 MLST on the CGE website (Carattoli et al. 2014). Blast comparisons of plasmids (accession numbers CP023365, CP023376, CP023382, KT186369 and AB021078) were performed with proksee (https://proksee.ca/).

## 3 Results

### 3.1 Isolates Identification

A total of 863 presumptive carbapenemase‐producing *E. coli* isolates were collected over the course of the study. Upon further analysis by MALDI‐TOF, only seven isolates were confirmed as *E. coli* (0.8%). MALDI‐TOF analysis of the 863 isolates revealed that the isolates belonged to 13 different genera (Table S1). The most frequently isolated genus was *Aeromonas*, which constituted 94.2% of the isolates (*n* = 813), followed by *Pseudomonas* (*n* = 15). The seven *E. coli* isolates originated from samples collected on the same day from upstream (*n* = 2) to downstream (*n* = 5) of the watershed. Data from *E. coli* and *Enterococci* enumeration (as indicators of faecal contamination) did not show any particular variation on that day (data not shown).

### 3.2 Antimicrobial Susceptibility Testing

The MICs were determined for the seven *E. coli* isolates. Out of the seven isolates, six of them (Table 1) presented a slightly reduced susceptibility to carbapenems with a NWT phenotype for ertapenem. All isolates were resistant to ampicillin and cefoxitin (MIC ≥ 64 mg/L) as well as to cefotaxime and ceftazidime (MIC ≥ 16 mg/L). The activity of these last two molecules was not restored by combination with clavulanic acid, which strongly suggests that the isolates produce a class C β‐lactamase (AmpC β‐lactamase) rather than a classical extended‐spectrum β‐lactamase (ESBL). All isolates were susceptible to all other antimicrobials tested.

**TABLE 1:** Minimal inhibitory concentration values for carbapenems of the six *E. coli* isolates.

	Ertapenem			Meropenem			Imipenem		
	MIC^a^	CB: 0.5^b^	T‐Ecoff: 0.03^c^	MIC	CB: 2–8	Ecoff: 0.06^d^	MIC	CB: 2–4	Ecoff: 0.5
LP12‐E4‐124	0.06	S	NWT	≤ 0.03	S	WT	0.5	S	WT
TK12‐E4‐120	0.06	S	NWT	0.06	S	WT	0.5	S	WT
TK12‐E4‐124	0.06	S	NWT	≤ 0.03	S	WT	0.5	S	WT
TK12‐E4‐125	0.06	S	NWT	≤ 0.03	S	WT	0.5	S	WT
TK12‐E4‐126	0.06	S	NWT	0.06	S	WT	0.25	S	WT
TK12‐E4‐127	0.06	S	NWT	0.06	S	WT	0.5	S	WT

^a^ MIC, minimal inhibitory concentration (mg/L).

^b^ CB, clinical breakpoint (mg/L); S, susceptible.

^c^ NWT, non‐wild type; T‐Ecoff, eucast tentative epidemiological cut‐off value (mg/L).

^d^ Ecoff, eucast epidemiological cut‐off value (mg/L); WT, wild type.

### 3.3 Isolate Typing

Whole genome sequencing of the six *E. coli* isolates with reduced carbapenem susceptibility revealed that they all share common properties (Table 2). All isolates had a predicted genome size of 4.7 Mb and belonged to phylogroup B1, serotype O8:H7 and ST196.

**TABLE 2:** Genetic characteristics of the six *E. coli* isolates with reduced susceptibility to carbapenems.

Isolate	Serotype	Phylogroup	ST	AMR genes	Plasmids	Genome size (bp)	Accession #
TK12‐E4‐127	O8:H7	B1	ST196	blaCMY‐2	IncFII, IncI1‐I(alpha)	4,783,179	JBKOPJ000000000
TK12‐E4‐126	O8:H7	B1	ST196	blaCMY‐2	IncFII, IncI1‐I(alpha)	4,785,539	JBKOPI000000000
TK12‐E4‐125	O8:H7	B1	ST196	blaCMY‐2	IncFII, IncI1‐I(alpha)	4,783,300	JBKOPH000000000
TK12‐E4‐120	O8:H7	B1	ST196	blaCMY‐2	IncFII, IncI1‐I(alpha)	4,768,833	JBKOPF000000000
TK12‐E4‐124	O8:H7	B1	ST196	blaCMY‐2	IncFII, IncI1‐I(alpha)	4,775,778	JBKOPG000000000
LP12‐E4‐124	O8:H7	B1	ST196	blaCMY‐2	IncFII, IncI1‐I(alpha)	4,782,423	JBKOPE000000000

**FIGURE 1:**
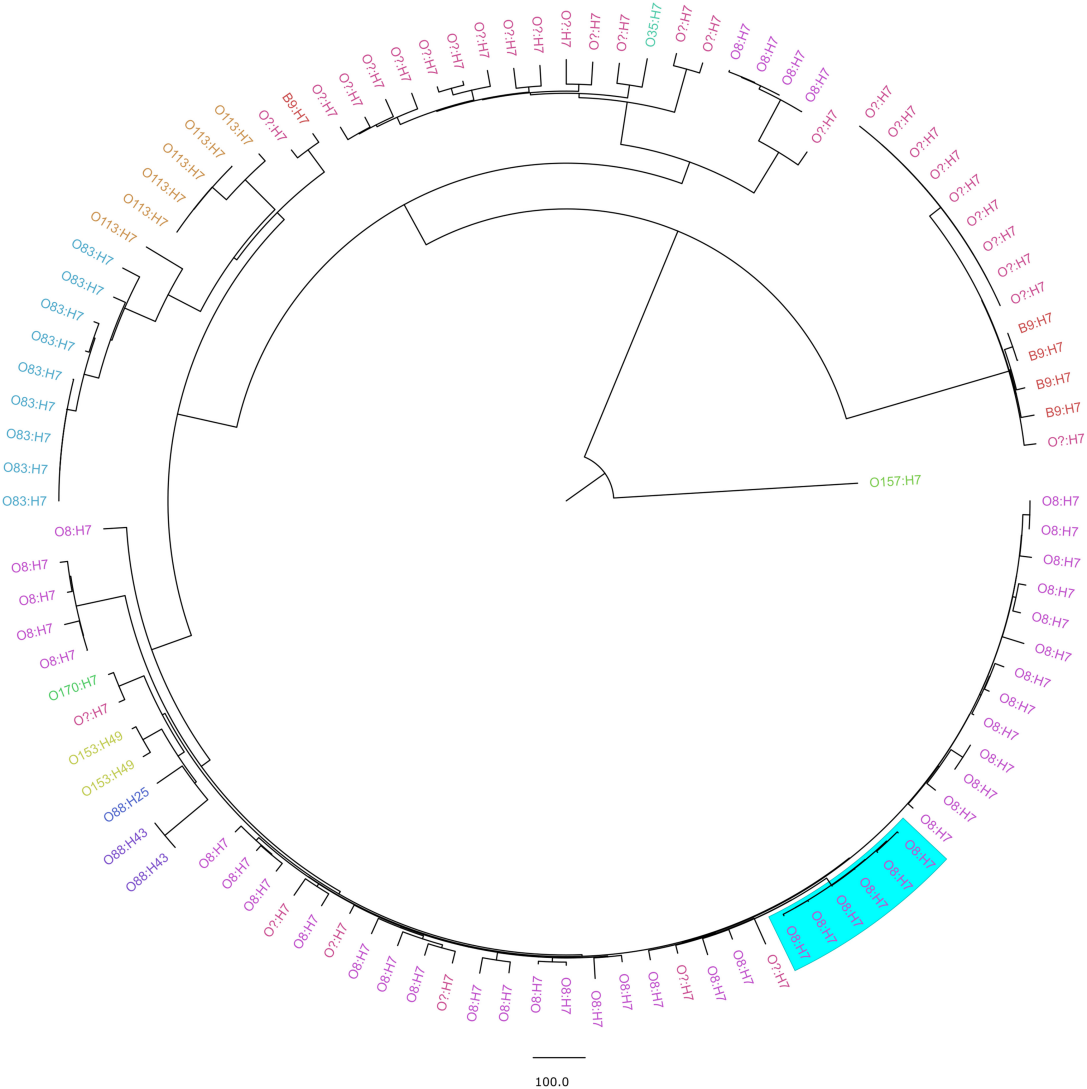
*E. coli* ST196 genomes (*n* = 103) comparison by cgMLST. Sequences and associated metadata were retrieved from Enterobase (*n* = 97). *E. coli* Sakai (O157:H7) was used as outgroup. The six isolates sequenced in this project are indicated by the blue rectangle. The isolates are coloured according to their serotype.

A phylogenetic comparison with other previously sequenced ST196 strains (Table S2) using core‐genome multi‐locus sequence typing (cgMLST) demonstrated that the six isolates were closely related to each other (as evidenced by the short branches, Figure 1). The average distance between the six isolates was four alleles, while it was on average 364 alleles between ST196 isolates and 135 alleles with the other O8:H7 strains of the dataset (Table S3). Furthermore, the genomes of the six isolates had an average ANI of 99.99% (Table S4). The close genetic proximity between the six isolates suggests they constitute a single clone.

Analysis of the virulome of the isolates using the *E. coli* virulence database from VirulenceFinder (version 28/11/2023) revealed that besides colicins, the isolates did not exhibit any specific virulence factors from the various *E. coli* pathogroups.

### 3.4 Genotypic Characterisation of AMR Determinants

The genome sequences of the six isolates were analysed using the ResFinder database to detect AMR genes. No carbapenemase‐associated gene was identified in any of the isolates. All six isolates exhibited a single resistance gene, *bla*_CMY‐2_, an AmpC beta‐lactamase (Table 2). In all isolates, the sequence of the *bla*_CMY‐2_ gene was 100% identical to the reference sequence (X91840).

**FIGURE 2:**
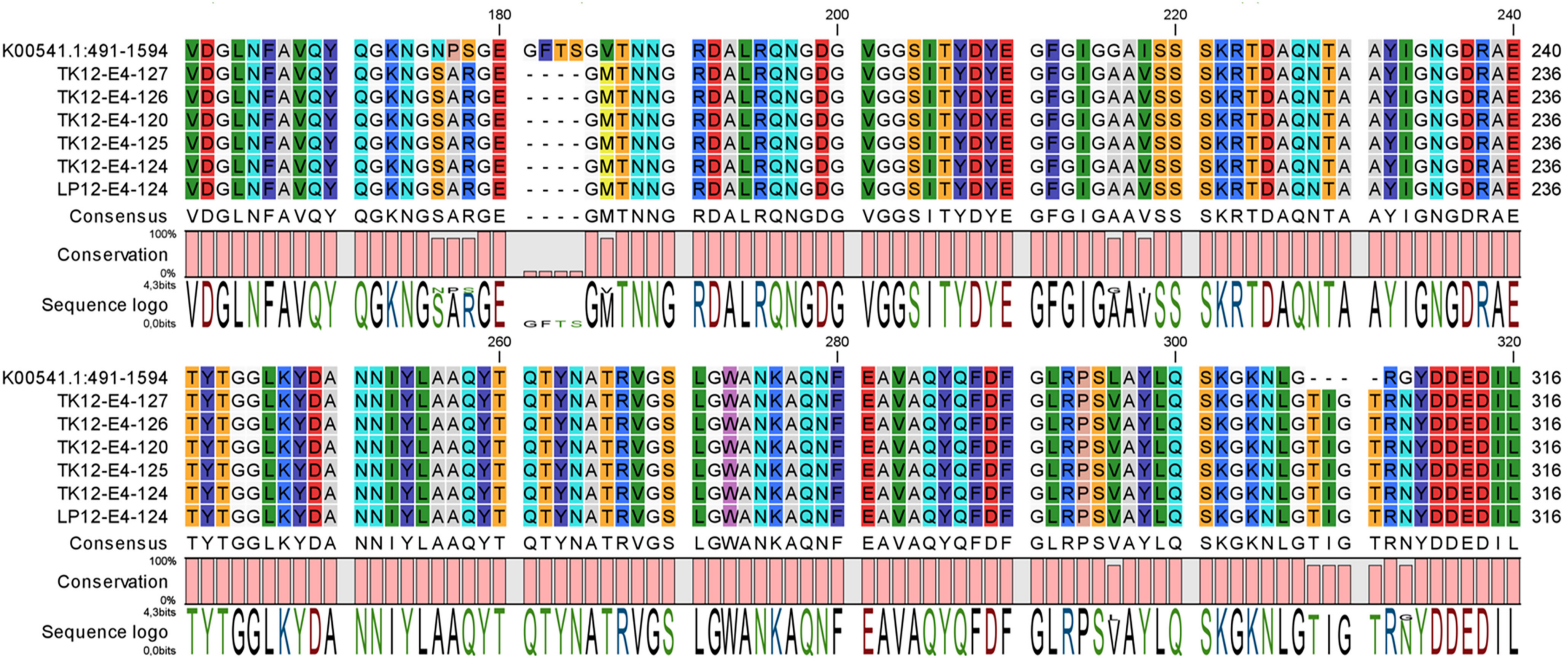
Protein sequence alignment of the L4–L7 region of the OmpC porin.

The integrity of the OmpC and OmpF porins coding genes was investigated. No mutation was found in the OmpF porin coding gene in any of the six isolates compared to the reference sequence (J01655.1). However, the sequence of the OmpC porin coding gene was altered in all six isolates. The sequence of the OmpC coding gene was identical in all six isolates and differed from the reference sequence (K00541.1) by 37 SNPs, a 12‐nucleotide deletion in position 544 (relative to the reference sequence) and a 12‐nucleotide insertion in position 921. This resulted in eight point‐mutations (two of them conservative) along the protein sequence, the deletion of four amino acids (AA) in position 181 (loop L4) and the insertion of four AA in position 308 (loop L7) (Figure 2).

Interestingly, all but seven of the ST196 strains in the genome dataset recovered from Enterobase exhibited the same *ompC* sequence. Two of these strains (both O8:H7) exhibited only one SNP compared with our isolates (which did not result in an amino acid change). The five other isolates (all of them O113:H7) had 13 SNPs compared to our isolates, seven of these, located in the L7 loop, resulted in three AA changes.

In order to identify the replicon carrying the *bla*_CMY‐2_ gene, we sequenced one of the isolates (isolate TK12‐E4‐120) using long‐read sequencing. MinION sequencing indicated that the *bla*_CMY‐2_ gene is located on an IncI1 plasmid belonging to clonal complex ST2 (CC‐2). The 94 kbp plasmid (p94) was circularised and contained 106 coding domain sequences, many of which were without known function.

**FIGURE 3:**
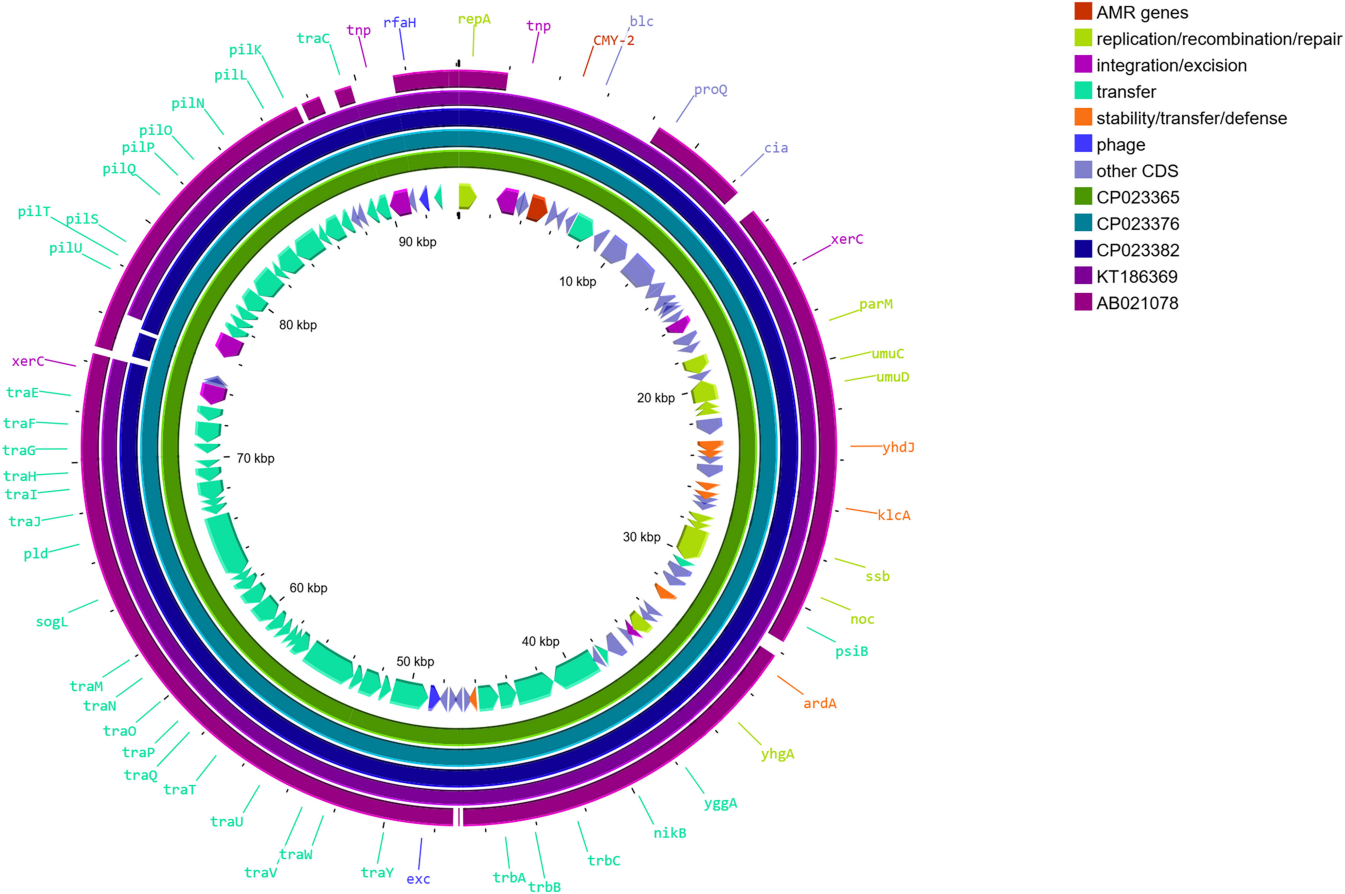
Circular representation of p94 plasmid. BLAST comparisons were performed with CMY‐2‐carrying IncI1 plasmids (accession numbers CP023365, CP023376, CP023382 and KT186369) and the IncI1 prototype plasmid P9 (AB021078). CDS are colour‐coded according to their function.

**FIGURE 4:**
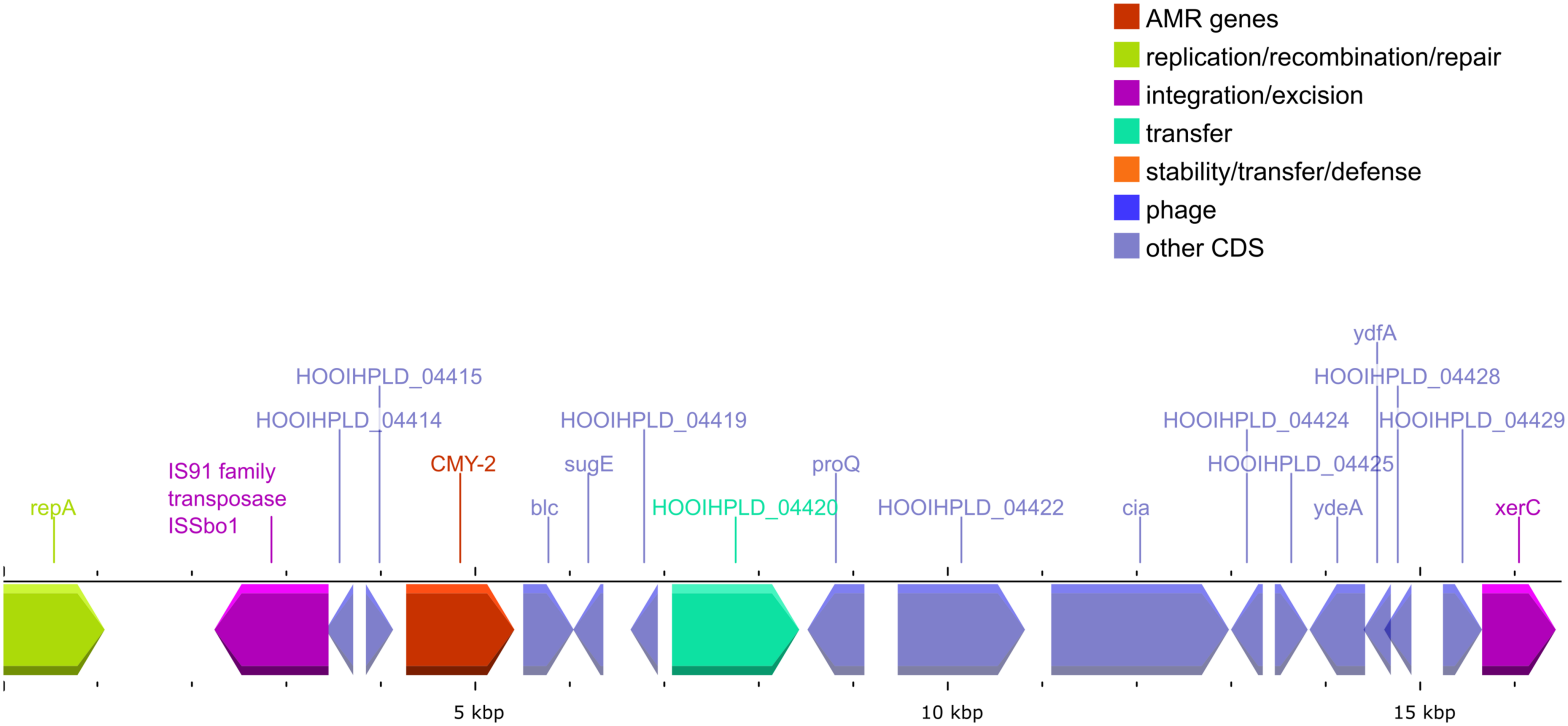
Representation of the mobile element carrying the CMY‐2 gene. CDS are colour‐coded according to their function as in Figure 3.

The p94 plasmid exhibited conjugative transfer regions (*tra*, *trb* and *pil* operons), genes involved in the inhibition of SOS response (*psiAB*) and genes involved in maintenance and stability (Figure 3). The *bla*_CMY‐2_ gene was located on a mobile genetic element flanked by an IS91 and *blc*‐*sugE* elements (Figure 4). Further analysis of the plasmid confirmed that it did not contain any other AMR genes. It also did not exhibit any known virulence factor besides colicin Ia.

A BLAST comparison of p94 with other CMY‐2‐carrying IncI1 ST2 plasmids (accession numbers CP023365, CP023376, CP023382 and KT186369) and the IncI1 prototype plasmid P9 (AB021078) (Carattoli et al. 2021) is shown in Figure 3. Plasmid p94 exhibited over 99% identity with the CMY‐2‐carrying IncI1 ST2 plasmids over 92–94 kb.

Within the ST196 genomes dataset (*n* = 97), only six additional strains were found to harbour the *bla*_CMY‐2_ gene, each representing diverse serotypes, sources and geographical origins. Notably, among these, two strains, both of serotype O8:H7, exhibited *bla*_CMY‐2_ as the sole identified resistance gene, and both strains were found to possess an IncI1 plasmid (although it should be noted that this does not conclusively confirm plasmid carriage of the gene). Two other strains, both characterised as O?:H7, were found to also carry the fosfomycin resistance gene *fosA7* and demonstrated the presence of both IncI1 and IncF plasmids. Between the final two strains, characterised as O8:H7 and O83:H7, multiple AMR genes were observed. Specifically, the O83:H7 strain was found to harbour an IncI1 plasmid, while the O8:H7 strain demonstrated the presence of both an IncY and an IncA/C plasmid.

In parallel, it appeared that a single clinical strain (O?:H7) from the dataset carried a carbapenemase gene (*bla*_KPC‐2_).

Further examination of the AMR genes in the dataset revealed that 42% of the strains (44 out of 104 strains) did not appear to carry any resistance gene, while 17% (*n* = 18) of the strains appeared to carry a single AMR gene (*bla*_CMY‐2_, *fosA7*, *bla*_CTX‐M‐15_, *aph(3′)‐IIa*, *mcr‐1* or *bla*_TEM‐1B_). All the other strains (*n* = 42, or 40%) carried at least two resistance genes and up to 17.

## 4 Discussion

Selection pressure due to the increased use of carbapenems as last‐resort treatment of infections due to multidrug‐resistant *Enterobacteriaceae* has contributed to the global emergence of carbapenem‐resistant strains. These strains typically produce a carbapenemase such as VIM, KPC, NDM, IMP and OXA‐48 (Nordmann et al. 2012). Although carbapenems are not licensed in veterinary sectors in Europe, third generation cephalosporins can be used (e.g., ceftiofur) in food‐producing animals with strict restrictions, which could promote the co‐selection of ESBL and carbapenem‐resistant strains. In Europe, the presence of carbapenem‐resistant bacteria has been reported in livestock, seafood, companion animals, wildlife, and the environment (Aberkane et al. 2015; Haenni et al. 2022; Ramírez‐Castillo et al. 2023).

Freshwater environments are potential hotspots for AMR exchange due to the combined presence of various sources of antimicrobial agents such as antibiotics, disinfectants and antifungals (Flores‐Vargas et al. 2021), together with complex microbial communities (biofilm and planktonic), including pathogens and microplastics that can serve as carriers for these bacteria and pose risks to aquatic biota and human health if they bypass the water treatment process.

The primary aim of this study was to explore whether existing microbiological drinking water quality monitoring practices could be leveraged for the detection of CRE in surface waters. Rather than providing a comprehensive survey of CRE or capturing low‐abundance resistance traits, our approach was designed to assess the feasibility of detecting contamination events using standard procedures. For this purpose, we adopted sampling volumes of up to 100 mL, as commonly used in drinking water surveillance (ISO 9308‐1), which also represented the practical upper limit for filtration given the turbidity and particulate content of surface waters that can clog membranes. In a surveillance context, given an appropriate isolation medium, the limitation of small sample volumes could be compensated for statistically by increasing the number of samples, thereby improving the reliability of the results.

In this study, we conducted a longitudinal investigation over an 18‐month period to detect carbapenem‐resistant *E. coli* in a rural French watershed impacted by effluents from three wastewater treatment plants and agricultural and aquaculture activities. In the absence of specific guidelines for detection of CRE in water, we followed those issued from the food chain, including the use of selective agar plates. Our results highlighted that ChromID Carba is not an adequate medium for isolating carbapenem‐resistant *E. coli* from freshwater samples, as only 0.8% of presumptive carbapenemase‐producing *E. coli* isolates were confirmed as *E. coli* and none of these was confirmed as carbapenemase‐producer.

The lack of specificity of the ChromID Carba medium towards Enterobacteriaceae had already been highlighted (Papadimitriou‐Olivgeris et al. 2014). Similarly, other commercial screening media have been described to detect non‐carbapenemase‐producing isolates showing reduced susceptibility to carbapenems (Girlich et al. 2013). Members of the genus *Aeromonas* are among the few microorganisms that can produce metallocarbapenemase activities. The CphA metallo‐beta‐lactamase of *A. hydrophila* is one of the most active carbapenemases known (Rossolini et al. 1995). It should be acknowledged that the *Aeromonas* isolates recovered are probably carbapenemase producers, although that was not verified in this study. We have, however, verified by whole genome sequencing that several *Aeromonas allosaccharophila* isolates recovered from the same samples carry a *cphA* gene (data not shown).

Surprisingly, the six confirmed *E. coli* isolates with reduced carbapenem susceptibility did not carry any carbapenemase gene. Instead, the reduced carbapenem susceptibility appeared to be due to an alternative mechanism that has already been extensively described in clinical strains (Poirel et al. 2004; van Boxtel et al. 2016; Nuramrum et al. 2017; Ye et al. 2018; Larkin et al. 2020). This mechanism involves the production of a beta‐lactamase combined with the loss of an outer membrane porin. It is postulated that the AmpC‐type beta‐lactamases could covalently bind the carbapenems sequestrated in the periplasm due to the loss of the porin, thus preventing them from reaching their target (Nordmann et al. 2012).

Although it has been reported that ChromID Carba was not well suited to detect carbapenem‐resistant Enterobacteriaceae with slightly reduced susceptibility to carbapenems (Pauly et al. 2020), other studies (Wilkinson et al. 2012) have nonetheless shown that Enterobacteriaceae producing ESBL and AmpC beta‐lactamases are sometimes able to grow on ChromID CARBA, which is in line with our results. The presence of carbapenemase producers in the samples probably enabled the isolates with slightly reduced susceptibility to grow, while they would not have been able to do so in their absence. This type of background flora is unusual in clinical and food samples. However, this poses a unique challenge for environmental samples.

Whole genome sequencing confirmed that the only resistance gene detected in our isolates is *bla*_CMY‐2_ associated with mutations in the porin OmpC. The AmpC beta‐lactamase CMY‐2 is a well‐known mechanism found in *E. coli* resistant to third‐generation cephalosporins (Carattoli et al. 2021). It is often associated with IncI1 or IncA/C plasmids (Hopkins et al. 2006; Rodríguez‐Navarro et al. 2020; Carattoli et al. 2021). The combined use of Illumina and ONT MinION sequencing allowed us to examine the genomes of the strains and fully characterise the plasmid carrying the CMY‐2 gene, which was identified on an IncI1 ST2 plasmid.

*E. coli* with AmpC beta‐lactamase CMY‐2 were found both in humans and in animals (Zamudio et al. 2024). In France, the presence of CMY‐2 has been established in broiler populations (Touzain et al. 2018; Baron et al. 2018) and in freshwater (Baron et al. 2020). Specifically, one study revealed that the *bla*_CMY‐2_ gene was exclusively linked with IncB/O/K/Z‐like or IncFIA/FIB plasmids (Touzain et al. 2018). Conversely, another study identified the CMY‐2 gene carried by an IncI1 ST12 plasmid, noteworthy for lacking any other detected resistance gene within these plasmids (Baron et al. 2018). The association of CMY‐2 with IncI1 ST12 plasmids extends beyond the borders of France and is widespread. This phenomenon is exemplified by its high‐frequency detection in poultry populations in Colombia (Castellanos et al. 2017). However, CMY‐2 carried by IncI1 ST2 plasmids appears to exhibit a distinct host range, suggesting a potential divergence in its epidemiological characteristics. CMY‐2‐producing IncI1 ST2 plasmids have been described with high frequency in human patients in Denmark (Carattoli et al. 2021; Hansen et al. 2016) and healthy dogs in France (Haenni, Saras, et al. 2014). They have also been associated with *E. coli* responsible for urinary tract infections in dogs in the UK (Wagner et al. 2017). It is suggested that this particular plasmid is endemic to the commensal *E. coli* population, especially in dogs (Wagner et al. 2017), selected by the frequent use of cephalosporins in pets.

Comparison of the p94 plasmid with other CMY‐2‐carrying IncI1 ST2 plasmids isolated between 2001 and 2011 showed little variation. The remarkable conservation of CMY‐2 carrying IncI1 ST2 plasmids over almost two decades evidences the extreme stability of this plasmid.

In itself, the mechanism of reduced carbapenem susceptibility observed here is not transferable because it results from two distinct phenomena: acquisition of a CMY‐2 carrying plasmid in a strain possessing a mutated porin. However, closer examination of the porin sequence in other *E. coli* strains revealed that all but seven of the 97 ST196 strains in our panel appear to have the same porin sequence. These strains have been sampled from diverse sources and geographical locations. Therefore, although it was not tested we can speculate that the introduction of the CMY‐2 carrying plasmid in any ST196 strain would most likely result in a reduced susceptibility to carbapenems.

## 5 Conclusion

There are many studies devoted to the spread of antibiotic resistance in hospitals, WWTPs and livestock effluents. However, it is critical to monitor downstream compartments. In particular, it seems crucial to prevent the dissemination of resistance to critically important antibiotics such as carbapenems into the environment, where new reservoirs could form. Antibiotic‐resistant bacteria should thus be actively monitored in the aquatic environment to detect their presence and to inform control and intervention strategies.

We used a culture‐based approach using a selective medium supplemented with antibiotics to detect the presence of viable *E. coli* having a reduced susceptibility to carbapenem. Over an 18‐month period, the 144 samples analysed yielded six *E. coli* isolates with a reduced carbapenem susceptibility, all isolated on the same day, suggesting a single contamination occurrence. The mechanism responsible for the reduced carbapenem susceptibility precludes horizontal transmission of carbapenem resistance. Furthermore, the absence of known virulence genes on these strains suggests a very limited associated public health significance. However, this illustrates the importance of monitoring the aquatic environment but also the methodological difficulties of such surveillance due to the poor performance of the isolation method.

## Author Contributions

**Laëtitia Le Devendec:** investigation, writing – review and editing. **Mai‐Lan Tran:** investigation, writing – review and editing. **Eric Jouy:** conceptualization, investigation, methodology, validation, writing – original draft. **Fabien Vorimore:** data curation, investigation, writing – review and editing. **Amandine Wilhelm:** investigation, writing – review and editing. **Benoit Gassilloud:** investigation, writing – review and editing. **Patrick Fach:** writing – review and editing. **Sandrine Baron:** conceptualization, funding acquisition, investigation, methodology, project administration, supervision, validation, writing – original draft. **Sabine Delannoy:** conceptualization, data curation, funding acquisition, investigation, methodology, project administration, supervision, validation, visualization, writing – original draft.

## Conflicts of Interest

The authors declare no conflicts of interest.

## Supporting information


**File S1.** Python script used during cgMLST analysis to remove samples with < 90% of alleles identified and loci identified in < 90% of samples.

**Table S1.** Genera of the isolates recovered from the CHROMID Carba plates as determined by MALDI‐TOF analysis.

**Table S2.** List of strains recovered from Enterobase with corresponding metadata.

**Table S3.** Allelic distance matrix between cgMLST profiles as calculated using cgmlst‐dists.

**Table S4.** ANI matrix of the six isolates sequenced in this study.

## Data Availability

Short and long raw reads were deposited in the NCBI SRA database under BioProject accession number PRJNA1199207 (SAMN45868978, SAMN45868979, SAMN45868980, SAMN45868981, SAMN45868982, SAMN45868983). Additionally, assembled genomes were deposited in GenBank under accession numbers JBKOPE000000000, JBKOPF000000000, JBKOPG000000000, JBKOPH000000000, JBKOPI000000000, and JBKOPJ000000000.
