# Correction to ‘On the Difficulty to Detect Carbapenem Resistance in the Environment: Characterisation of *Escherichia coli* With Reduced Carbapenem Susceptibility Isolated in a French River’

**DOI:** 10.1111/1758-2229.70378

**Published:** 2026-06-10

Le Devendec, L. , M.‐L. Tran , E. Jouy , et al. 2025. “On the Difficulty to Detect Carbapenem Resistance in the Environment: Characterisation of *Escherichia coli* With Reduced Carbapenem Susceptibility Isolated in a French River.” Environmental Microbiology Reports 17, no. 4: e70162. 10.1111/1758-2229.70162.40692201 PMC12280049

The authors' first names and surnames in the author byline were displayed incorrectly. The author byline should be corrected to: Laëtitia Le Devendec, Mai‐Lan Tran, Eric Jouy, Fabien Vorimore, Amandine Wilhelm, Benoit Gassilloud, Patrick Fach, Sandrine Baron, and Sabine Delannoy.

The online version of the article has been corrected.

We apologise for this error.

