# Supplementary material for: On the Difficulty to Detect Carbapenem Resistance in the Environment: Characterisation of Escherichia coli With Reduced Carbapenem Susceptibility Isolated in a French River
Source: Environ Microbiol Rep. 2025 Jul 21;17(4):e70162. doi: 10.1111/1758-2229.70162 (PMC12280049; doi:10.1111/1758-2229.70162)
Supplement: Supplementary file 1 — File S1. Python script used during cgMLST analysis to remove samples with < 90% of alleles identified and loci identified in < 90% of samples. [file EMI4-17-e70162-s001.pdf]

```python
#!/usr/bin/env python

import argparse
import os
import pandas as pd

def main(input_file, output_folder, individual_value, character_value):
    # Read the input file into a DataFrame
    df = pd.read_csv(input_file, delimiter='\t')

    # Count the number of characters in the original dataframe
    num_characters_original = len(df.columns) - 1

    # Check for numerical values in each individual and remove those with less than the specified individual value
    individuals_to_remove = []
    for index, row in df.iterrows():
        num_values = sum(pd.to_numeric(row[1:], errors='coerce').notnull())
        total_values = len(row) - 1
        if num_values / total_values < individual_value:
            individuals_to_remove.append(row[0])

    df_clean = df[~df.iloc[:, 0].isin(individuals_to_remove)]

    # Save removed individuals to a new file
    removed_individuals_df = df[df.iloc[:, 0].isin(individuals_to_remove)]
    removed_individuals_df.to_csv(os.path.join(output_folder, 'indiv_out.tsv'), sep='\t', index=False)

    # Check for numerical values in each character and remove those with less than the specified character value
    characters_to_remove = []
    for column in df_clean.columns[1:]:
        num_values = pd.to_numeric(df_clean[column], errors='coerce').notnull().sum()
        total_values = len(df_clean)
        if num_values / total_values < character_value:
            characters_to_remove.append(column)

    df_clean = df_clean.drop(columns=characters_to_remove)

    # Save removed characters to a file
    with open(os.path.join(output_folder, 'alleles_out.txt'), 'w') as f:
        f.write('\n'.join(characters_to_remove))

    # Save the cleaned DataFrame to a new file
    df_clean.to_csv(os.path.join(output_folder, 'results_alleles_clean.tsv'), sep='\t', index=False)

    # Count the number of characters in the cleaned dataframe
    num_characters_cleaned = len(df_clean.columns) - 1

    # Write the character counts to the output file
    with open(os.path.join(output_folder, 'cgMLST-stat.txt'), 'w') as f:
        f.write(f"Number of characters in the original dataframe: {num_characters_original}\n")
        f.write(f"Number of characters in the cleaned dataframe: {num_characters_cleaned}\n")

if __name__ == '__main__':
    parser = argparse.ArgumentParser(description='Clean dataframe and output statistics.')
    parser.add_argument('-i', '--input', type=str, help='Path and name of the input file')
    parser.add_argument('-o', '--output', type=str, help='Path of the output folder')
    parser.add_argument('-c', '--individual-value', type=float, help='Value to check individuals')
    parser.add_argument('-a', '--character-value', type=float, help='Value to check characters')
    args = parser.parse_args()

    main(args.input, args.output, args.individual_value, args.character_value)
```

```
#To run this script, execute it from the command line, providing the appropriate arguments. For example:
#   python script.py -i input_folder/results_alleles.tsv -o output_folder -c 0.9 -a 0.9

#Make sure to replace script.py with the actual name of the Python script file, input_folder/results_alleles.tsv with the path and name of the input file, output_folder with the path to the desired output folder, and 0.9 with the chosen values for checking individuals and characters.

#This script allows to pass the input file path and name using -i, the output folder path using -o, the value to check individuals using -c, and the value to check characters using -a. The output files will be saved in the specified output folder.
```
