# Supplementary material for: On the Difficulty to Detect Carbapenem Resistance in the Environment: Characterisation of Escherichia coli With Reduced Carbapenem Susceptibility Isolated in a French River
Source: Environ Microbiol Rep. 2025 Jul 21;17(4):e70162. doi: 10.1111/1758-2229.70162 (PMC12280049; doi:10.1111/1758-2229.70162)
Supplement: Supplementary file 2 — Table S1. Genera of the isolates recovered from the CHROMID Carba plates as determined by MALDI‐TOF analysis. [file EMI4-17-e70162-s005.pdf]

Table S1: Genera of the isolates recovered from the CHROMID Carba plates as determined by MALDI-TOF analysis

Genus	number
Aeromonas	813
Pseudomonas	15
Escherichia	7
Stenotrophomonas	8
Micrococcus	5
Staphylococcus	4
Enterococcus	3
Carnobacterium	2
Bacillus	2
Arcobacter	1
Citrobacter	1
Acinetobacter	1
Streptococcus	1
**Total**	**863**
